# Characteristics of contralateral carcinomas in patients with differentiated thyroid cancer larger than 1 cm

**DOI:** 10.1007/s00423-016-1393-4

**Published:** 2016-03-24

**Authors:** Lutske Lodewijk, Wouter P. Kluijfhout, Jakob W. Kist, Inge Stegeman, John T. M. Plukker, Els J. Nieveen van Dijkum, H. Jaap Bonjer, Nicole D. Bouvy, Abbey Schepers, Johannes H. W. de Wilt, Romana T. Netea-Maier, Jos A. van der Hage, Jacobus W. A. Burger, Gavin Ho, Wayne S. Lee, Wen T. Shen, Anna Aronova, Rasa Zarnegar, Cassandre Benay, Elliot J. Mitmaker, Mark S. Sywak, Ahmad M. Aniss, Schelto Kruijff, Benjamin James, Raymon H. Grogan, Laurent Brunaud, Guillaume Hoch, Chiara Pandolfi, Daniel T. Ruan, Michael D. Jones, Marlon A. Guerrero, Gerlof D. Valk, Inne H. M. Borel Rinkes, Menno R. Vriens

**Affiliations:** University Medical Center Utrecht, Heidelberglaan 100, 3584 CX Utrecht, The Netherlands; University Medical Center Groningen, Hanzeplein 1, 9700 RB Groningen, The Netherlands; Academic Medical Center Amsterdam, Meibergdreef 9, 1105 AZ Amsterdam, The Netherlands; VU Medical Center, De Boelelaan 1117, 1081 HZ Amsterdam, The Netherlands; Maastricht University Medical Center, P. Debyelaan 25, 6229 HX Maastricht, The Netherlands; Leiden University Medical Center, Albinusdreef 2, 2333 ZA Leiden, The Netherlands; Radboud University Medical Center, Geert Grooteplein-Zuid 10, 6525 GA Nijmegen, The Netherlands; Netherlands Cancer Institute, Plesmanlaan 121 – 123, 1066 CX Amsterdam, The Netherlands; Erasmus Medical Center, ‘s-Gravendijkwal 230, 3015 CE Rotterdam, The Netherlands; University of California San Francisco Medical Center, 505 Parnassus Ave, San Francisco, CA 94143 USA; Weill Cornell Medical Center, 525 E 68th St, New York, NY 10065 USA; McGill University Health Centre, 1650 Cedar Avenue, Montreal, Quebec H3G 1A4 Canada; Endocrine Surgery Unit, University of Sydney, Camperdown, NSW 2006 Australia; The University of Chicago Medical Center, 5841 S Maryland Ave, Chicago, IL 60637 USA; Centre Hospitalier Universitaire de Nancy, 29 Avenue du Maréchal de Lattre de Tassigny, 54000 Nancy, France; Brigham and Women’s Hospital, 75 Francis St, Boston, MA 02115 USA; The University of Arizona Medical Center, 3838 N Campbell Ave, Tucson, AZ 85719 USA; Department of Surgery (G.04.228), University Medical Center Utrecht, P.O. Box 85500, 3508 GA Utrecht, The Netherlands

**Keywords:** Differentiated thyroid carcinoma, Contralateral carcinoma, Papillary microcarcinoma, Surgical strategy

## Abstract

**Purpose:**

Traditionally, total thyroidectomy has been advocated for patients with tumors larger than 1 cm. However, according to the ATA and NCCN guidelines (2015, USA), patients with tumors up to 4 cm are now eligible for lobectomy. A rationale for adhering to total thyroidectomy might be the presence of contralateral carcinomas. The purpose of this study was to describe the characteristics of contralateral carcinomas in patients with differentiated thyroid cancer (DTC) larger than 1 cm.

**Methods:**

A retrospective study was performed including patients from 17 centers in 5 countries. Adults diagnosed with DTC stage T1b-T3 N0-1a M0 who all underwent a total thyroidectomy were included. The primary endpoint was the presence of a contralateral carcinoma.

**Results:**

A total of 1313 patients were included, of whom 426 (32 %) had a contralateral carcinoma. The contralateral carcinomas consisted of 288 (67 %) papillary thyroid carcinomas (PTC), 124 (30 %) follicular variant of a papillary thyroid carcinoma (FvPTC), 5 (1 %) follicular thyroid carcinomas (FTC), and 3 (1 %) Hürthle cell carcinomas (HTC). Ipsilateral multifocality was strongly associated with the presence of contralateral carcinomas (OR 2.62). Of all contralateral carcinomas, 82 % were ≤10 mm and of those 99 % were PTC or FvPTC. Even if the primary tumor was a FTC or HTC, the contralateral carcinoma was (Fv)PTC in 92 % of cases.

**Conclusions:**

This international multicenter study performed on patients with DTC larger than 1 cm shows that contralateral carcinomas occur in one third of patients and, independently of primary tumor subtype, predominantly consist of microPTC.

## Introduction

Differentiated thyroid cancer (DTC) is the most common endocrine malignancy and its incidence is rising. The prognosis is excellent with 10-year survival rates over 90 % irrespective of the stage of disease [1]. Until recently, in western countries, treatment of DTC was similar for all stages of macroDTC (DTC larger than 1 cm): total thyroidectomy followed by radioactive iodine ablation (RAI) therapy [2, 3]. However, in the last decade, single-center studies performed in large-volume centers showed no significant differences in recurrence and survival rates in patients diagnosed with macroDTC, who were either treated with lobectomy or total thyroidectomy [4–7]. This has evoked a new discussion about the optimal extent of surgery, whereby according to the ATA and NCCN guidelines (2015, USA), patients with tumors up to 4 cm are now eligible for lobectomy [8, 9].

Traditional arguments for adhering to total thyroidectomy are the presence of contralateral carcinomas, the ability to perform RAI and the use of thyroglobulin as a follow-up marker. There is, however, increasing support for more selective use of RAI [9–11]. Contralateral carcinomas are reported in up to 44 % of patients with DTC [12]. Supporters of total thyroidectomy argue that contralateral carcinomas could affect disease recurrence and survival [12–15]. Interestingly, these data are mainly based on patients with microDTC (DTC smaller than 1 cm) and data on the incidence of contralateral carcinomas in macroDTC is currently scarce [16–21].

We, therefore, aimed to describe the incidence and the characteristics of contralateral carcinomas, and subsequently assess determinants correlating with the presence of contralateral carcinomas in patients with macroDTC.

## Patients and methods

### Patients

We conducted a descriptive, retrospective, cross-sectional, multicenter study in a total of 17 centers in 5 countries. Patients who underwent a total thyroidectomy for DTC, either in one or two stages, who were operated between January 2000 and December 2012 and aged ≥18 years were included. Indication for a completion thyroidectomy was confirmation of DTC larger than 1 cm in the histologic examination of the lobectomy specimen.

We specifically selected the patients for whom the discussion about the extent of surgery is most relevant. The TNM stages that were included for the different histological subtypes were based on the currently recruiting study of Mallick et al. [10]. This study investigates whether in a subgroup of low-risk patients ablation can be omitted, without compromising recurrence or survival rates. This concerns patients with a papillary thyroid carcinoma (PTC) including, follicular variant of papillary thyroid carcinoma (FvPTC) with stage pT1b-T2-T3, N0-N1a-Nx and patients with a follicular thyroid carcinoma (FTC) or a Hürthle cell carcinoma (HTC) stages pT1b-T2, N0-N1a-Nx. The TNM classification from the 7th edition of the AJCC cancer staging manual was used [22].

In Dutch University Medical Centers, all consecutive patients who were operated between 2000 and 2012 were included since these were only limited numbers. In the high-volume international centers, 150 patients were randomly generated from a list that included all patients that fulfilled inclusion criteria who were operated between 2000 and 2012. Cases were selected by creating a list of numbers generated by randomization software. Pathologic staging was performed according to the AJCC cancer staging manual. In the seven participating Dutch University Medical Centers, data entry was performed by the same researcher (WPK). Outside The Netherlands, data were collected by a local investigator, using a well-defined data entry manual to ensure homogeneous input. The study was approved by the institutional review board of the University Medical Center Utrecht (The Netherlands) and in other centers if required.

### Characteristics of contralateral carcinomas

The following characteristics of the contralateral carcinomas were collected: size, histological subtype, and contralateral multifocality.

### Determinants associated with contralateral disease

After performing a pilot study in 30 patients from the UMC Utrecht and by reviewing the recent literature, 13 determinants were selected [17, 20, 23–25]. Determinants included sex, age at diagnosis, size on ultrasound of primary tumor, Bethesda classification of the primary tumor, postoperative N-stage, size of the contralateral lobe on pathology (PA), size of the primary tumor, histological subtype of the primary tumor, multifocality in the lobe of the primary tumor (ipsilateral multifocality), angioinvasion, capsular invasion (cells invading the capsule of the tumor), extrathyroidal growth, and surgical resection margins of the primary tumor (defined by evaluating resection margins at pathology). Data were collected from chart reviews, cytology reports of fine-needle aspiration (FNA), reports of preoperative ultrasound, and the histology reports.

### Statistical analysis

All continuous variables were tested for linear association with the outcome, and in the case of non-linearity, the variable was categorized in clinically relevant groups [26]. The possible determinants were assessed for patients with and without contralateral carcinoma, and univariate regression analysis and multivariate regression analysis were performed. Variables with a *p* value <0.1 in the univariate regression analysis were selected for multivariate analysis. A *p* value <0.05 was considered statistically significant. All statistical analyses were performed using SPSS version 22 (SPSS Inc., Chicago, IL).

## Results

### Patients

In total, we included 1313 patients in 17 centers (Table 1). The mean age at time of surgery was 47.4 years (SD 14.5), and 967 (74 %) patients were female. Total thyroidectomy as primary surgical intervention was performed in 961 (73 %) patients, whereas 352 (27 %) patients initially had a lobectomy followed by completion thyroidectomy. Central lymph node dissection was not standard of care but was performed dependent of the presence of suspicious lymph nodes on preoperative ultrasound, preference of the surgeon and the clinic. The histological subtype of the primary tumor was PTC in 794 patients (61 %), FvPTC in 354 (27 %), FTC in 116 (9 %), and HTC in 38 (3 %). Unilateral tumor multifocality was seen in 277 (21 %) patients, and 269 (22 %) had central lymph node metastases. Capsular invasion, angioinvasion, and extra-thyroidal growth were found in 415 (41 %), 247 (22 %), and 231 (19 %) cases, respectively.

**Table 1:** Distribution of number of included patients per center

Country	Hospital	Number of patients
The Netherlands	UMC Utrecht	40
	UMC Groningen	50
	Leiden UMC	50
	Radboud UMC	31
	Maastricht UMC+	15
	Erasmus UMC	49
	VU Medical Center	26
	Amsterdam Medical Center	26
	Antonie van Leeuwenhoek Hospital	23
USA	University of California San Francisco	106
	Weill Cornell Medical College	106
	University of Chicago	128
	Brigham and Women’s Hospital	145
	The University of Arizona Medical Center	77
Canada	McGill University Health Center	99
France	Centre Hospitalier Universitaire de Nancy	137
Australia	Royal North Shore Hospital	205

### Characteristics of contralateral carcinomas

The overall rate of contralateral carcinomas was 32 % (Table 2). The majority of contralateral carcinomas were PTC (*N* = 284; 69 %) or FvPTC (*N* = 123; 30 %), while only a few were FTC (*N* = 4; 1 %) and HTC (*N* = 3; 1 %) (Table 3). The median size of the contralateral carcinomas was 4 mm (IQR 2–9 mm). If the primary tumor was non-(Fv)PTC, so FTC or HTC, the contralateral carcinoma was (Fv)PTC in 92 % of the cases. Sixty percent of the contralateral tumors were 5 mm or smaller, 21 % were between 6 and 10 mm, and 18 % were larger than 10 mm. Of the 82 % of tumors sized 10 mm or smaller, 99 % were PTC or FvPTC. Six out of the total of eight contralateral FTCs or HTCs were 10 mm or larger (Table 4).

**Table 2:** Descriptive statistics for the study population

Determinants	Number	Contralateral carcinoma +	OR (95 % CI) univariate analyses	*p* value	OR (95 % CI) multivariate analyses	*p* value
*N*	1313	426 (32 %)				
Sex						
Female	967 (74 %)	320 (75 %)	0.89 (0.69–1.14)	0.40		
Male	346 (26 %)	106 (25 %)				
Age			1.01 (1.00–1.02)	0.07	1.02 (1.01–1.04)	0.01
≤45	579 (44 %)	182 (43 %)				
≥45	734 (56 %)	244 (57 %)				
Size primary tumor US (mm)			1.00 (1.00–1.01)	0.41		
<11	32 (3 %)	13 (4 %)				
11–20	384 (37 %)	132 (39 %)				
21–30	325 (31 %)	87 (25 %)				
31–40	148 (14 %)	49 (14 %)				
>40	162 (15 %)	61 (18 %)				
Missing	262					
FNA (Bethesda)						
1	57 (5 %)	15 (4 %)				
2	91 (8 %)	34 (9 %)	0.61 (0.33)	0.11	0.58 (0.21)	0.30
3	85 (8 %)	25 (7 %)	1.01 (0.64)	0.96	0.83 (0.35)	0.68
4	213 (19 %)	55 (15 %)	0.71 (0.43)	0.18	0.89 (0.37)	0.79
5	205 (19 %)	63 (18 %)	0.59 (0.41)	0.00	0.68 (0.37)	0.22
6	456 (41 %)	169 (47 %)	0.75 (0.53)	0.12	0.93 (0.50)	0.81
Missing	206					
Nodal status						
N0	979 (78 %)	397 (73 %)	1.56 (1.18–2.07)	0.00	1.89 (1.07–3.34)	0.03
N1a	269 (22 %)	109 (27 %)				
Missing	65					
Size primary tumor PA (mm)			1.01 (1.00–1.02)	0.07	1.02 (1.00–1.05)	0.03
11–20	618 (47 %)	197 (46 %)				
21–30	370 (28 %)	115 (27 %)				
31–40	163 (12 %)	49 (12 %)				
>40	162 (12 %)	65 (15 %)				
Angio-invasion						
No	901 (78 %)	298 (79 %)	0.93 (0.69–1.26)	0.66		
Yes	247 (22 %)	78 (21 %)				
Missing	165					
Capsular invasion						
No	594 (59 %)	187 (60 %)	0.93 (0.71–1.22)	0.59		
Yes	415 (41 %)	124 (40 %)				
Missing	304					
Extra-thyroidal growth						
No	1006 (81 %)	307 (77 %) 94 (23 %)	1.56 (1.16–2.10)	0.00	1.02 (0.53–1.93)	0.96
Yes	231 (19 %)					
Missing	76					
Negative margins						
No	374 (29 %)	121 (30 %)	0.99 (0.77–1.28)	0.95		
Yes	898 (71 %)	289 (70 %)				
Missing	41					
Multifocality						
No	1033 (79 %)	285 (67 %)	2.64 (2.01–3.47)	0.00	2.62 (1.60–4.29)	0.00
Yes	277 (21 %)	139 (33 %)				
Missing	3					
Subtype carcinoma						
PTC	794 (61 %)	280 (67 %)				
FvPTC	354 (27 %)	113 (27 %)	0.86 (0.66)	0.27	0.58 (0.35)	0.04
FTC	116 (9 %)	20 (5 %)	0.38 (0.23)	0.00	0.54 (0.22)	0.18
HTC	38 (3 %)	6 (1 %)	0.34 (0.14)	0.02	0.16 (0.03)	0.02
Missing	11					
Size contralateral lobe (mm)			1.00 (1.00–1.00)	0.59		
<10	261 (24 %)	68 (20 %)				
>10–<15	254 (24 %)	85 (25 %)				
>15–<25	262 (24 %)	81 (23 %)				
>25	299 (28 %)	113 (33 %)				
Missing	237					

Number (*N*) of patients with a contralateral carcinoma is shown for each determinant. Odds–Ratios (OR) and *p* values are shown for the uni- and multivariate analyses

*US* ultrasonography, *FNA* fine-needle aspiration, *PA* pathology

**Table 3:** Histological subtype of the primary tumors versus the histological subtype of the contralateral tumor. Missing: *n* = 14 (3 %)

	Subtype of primary tumor					
Subtype of contralateral tumor		PTC	FvPTC	FTC	HTC	Total
	PTC	244 (88 %)	25 (23 %)	13 (65 %)	2 (33 %)	284 (69 %)
	FvPTC	30 (11 %)	84 (76 %)	6 (30 %)	3 (50 %)	123 (30 %)
	FTC	1 (0 %)	2 (2 %)	1 (5 %)	0 (0 %)	4 (1 %)
	HTC	2 (1 %)	0 (0 %)	0 (0 %)	1 (17 %)	3 (1 %)
	Total	277 (100 %)	111 (100 %)	20 (100 %)	6 (100 %)	414 (100 %)

**Table 4:** Size of the contralateral tumor versus the histological subtype of the contralateral tumors. Missing: *n* = 34 (8 %)

	Size of contralateral tumor				
Subtype of contralateral tumor		≤5 mm	6–10 mm	>10 mm	Total
	PTC	169 (71 %)	54 (64 %)	45 (63 %)	268 (68 %)
	FvPTC	68 (29 %)	29 (35 %)	21 (29 %)	118 (30 %)
	FTC	0 (0 %)	1 (1 %)	4 (6 %)	5 (1 %)
	HTC	1 (0 %)	0 (0 %)	2 (3 %)	3 (1 %)
	Total	238 (100 %)	84 (100 %)	72 (100 %)	394 (100 %)

Ipsilateral multifocality was most frequent when the primary tumor was PTC (23 %), followed by FvPTC (20 %), HTC (18 %), and FTC (9 %). The histological subtype of the ipsilateral tumors was PTC or FvPTC in 99 % of patients (data not shown).

### Determinants associated with contralateral disease

Based on the presence of contralateral carcinomas, univariate analysis of possible determinants was performed (Table 2). Contralateral carcinoma was significantly more frequent in patients with N1a nodal metastasis (OR 1.56 95 % CI 1.18–2.07), in tumors with extra-thyroidal growth (OR 1.56 95 % CI 1.16–2.10), and when ipsilateral multifocality was found (OR 2.64 95 % CI 2.01–3.47). When the histologic subtype of the primary tumor was a FTC or a HTC, the likelihood that a contralateral carcinoma was present decreased significantly (FTC: OR 0.38 95 % CI 0.23–0.63; HTC: OR 0.34 95 % CI 0.14–0.83). In multivariate analysis, ipsilateral multifocality (OR 2.62 95 % CI 1.60–4.29) and lymph node metastasis (OR 1.89 95 % CI 1.07–3.34) were strongly correlated with the occurrence of contralateral carcinoma(s). Furthermore, when the primary carcinomas were FvPTC, FTC, or HTC, there was a reversed correlation with the occurrence of contralateral carcinomas (FvPTC: OR 0.58 95 % CI 0.35–0.97; FTC: OR 0.54 95 % CI 0.22–1.33; HTC: OR 0.16 95 % CI 0.03–0.77). The other investigated determinants, sex, age, size primary tumor (US or PA), angioinvasion, capsular invasion, negative resection margins, and size of the contralateral lobe, did not correlate with the presence of contralateral carcinomas.

## Discussion

In this international multicenter study, the incidence and characteristics of contralateral carcinomas were investigated in a large cohort of patients with primary macroDTC. The rate of contralateral malignancies was 32 %, and the dominant histological subtype of the contralateral carcinomas was PTC or FvPTC (94 %). Median size was 4 mm and 82 % of carcinomas was <1 cm. No correlation between histological subtype of the primary tumor and the subtype of the contralateral tumor was found. Multifocality in the lobe of the primary tumor had the strongest association with contralateral carcinoma in multivariate analysis with an OR of 2.62.

The rate of contralateral carcinomas is in agreement with current literature that reports rates between 17 and 43 %. Most of these studies focused on contralateral carcinomas in primary papillary thyroid microcarcinomas (microPTC) [16, 17, 19–21], or had limited patient numbers, failed to report clear in- and exclusion criteria, or excluded patients with FTC [25, 27–29]. In contrast, our study investigated contralateral carcinoma in a large, well-described, and clinically relevant cohort, in which primary tumors were macroDTC. In our study, the rate of contralateral carcinomas was higher in PTC and FvPTC compared to FTC and HTC, 34 versus 17 %. This is in line with a study by Machens et al., who found significantly more tumor multifocality in patients with PTC versus FTC [30].

In our study, 82 % of all contralateral carcinomas were microPTC. Based on several other studies, the clinical relevance of microPTCs can be questioned. In an observational trial performed in Japan, including 1235 patients with primary microPTC, tumor progression of more than 3 mm was noticed in only 8.0 % of patients, novel nodal metastasis developed in 3.8 %, and only 6.8 % developed into clinical disease after 10 years of follow-up. Eventually, only 15 % of patients underwent surgery [11, 31]. These low progression rates show that these primary microPTCs rarely develop into clinically significant thyroid carcinomas. Our study described contralateral microcarcinomas, while this study addressed primary microPTC, and currently, it is unknown whether the natural course of the *primary* microPTC differs from those of contralateral microPTC. However, this is indirectly investigated by analyzing recurrence rates in the contralateral lobe in studies where DTC is treated by lobectomy. In a study with up to 20 years of follow-up comparing patients with microPTC treated with lobectomy versus treatment with total thyroidectomy, no difference in overall survival or in recurrence rates were found [5]. One might assume that in the remaining thyroid lobe, similar rates of contralateral carcinomas were present as in our population. Furthermore, from autopsy reports, it is known that when thyroid glands are thoroughly examined, malignancy rates of up to 36 % are found which is similar to our contralateral carcinoma rate [1, 32]. Altogether, the clinical relevance of 82 % of the contralateral carcinomas found in our study is questionable.

Currently, there is no consensus whether multifocal carcinomas arise as a result of true multicentricity or intrathyroidal spread of a primary tumor, as it is underlined by the report of the European Society of Endocrine Surgeons 2013 [33]. In our study, no correlation was found between the histological subtype of the primary tumor and that of the contralateral carcinoma. Moreover, even when the primary tumor was of follicular or Hürthle cell origin, 99 % of the contralateral carcinomas was PTC. This suggests that true multicentricity is more likely than intrathyroidal spread.

Patients with DTC have an excellent 10-year overall survival, but they do suffer from a relatively low quality of life (QoL) in comparison with other cancers, such as breast or colorectal cancer [34, 35]. This decreased QoL is expressed in several adverse physical, psychological, social, and spiritual challenges [34]. After RAI, patients had significantly more complaints of hypo- or hyperthyroidism, resulting in a decreased QoL[35]. Therefore, one of the factors to improve QoL of macroDTC patients is to pursue normal thyroid hormone homeostasis by performing parenchyma-sparing operations. After thyroid lobectomy, hormone replacement might be necessary in 10 to 50 % of patients; this is highly correlated to the TSH level and presence of microsomal antibodies. In patients with low TSH level (<2.5 mIU/L) and without microsomal antibodies, the risk to become hormone replacement dependent is only 7 %. Furthermore, after lobectomy, patients needed a lower dose of levothyroxine and less adjustment steps to become euthyroid [36–38]. Therefore, QoL might improve by performing a lobectomy instead of total thyroidectomy.

As the discussion continues, we believe treatment of DTC will become more and more a patient-tailored matter, in which the pros and cons of lobectomy or total thyroidectomy must be weighed one by one, based on existing evidence and discussed with the patient. The three main arguments in favor of total thyroidectomy are the ability to perform RAI, the use of thyroglobulin as a follow-up marker, and the high rate of contralateral carcinomas. Arguments in favor of lobectomy are reduced complication risk, especially recurrent laryngeal nerve injury and persisting hypocalcaemia, reduced risk of hypothyroidism, and no risk of complications from RAI. Taken the abovementioned arguments into account, we question whether, in case the ultrasound of the contralateral lobe does not show suspicious lesions, the possible presence of microPTC should be an argument in favor of total thyroidectomy.

## Conclusion

This international multicenter study is the largest study performed on patients with macroDTC and confirms that, in patients with macroDTC, the rate of contralateral carcinomas is 32 %. This study shows that these contralateral carcinomas predominantly consist of microPTC.
